# Supplementary material for: The interactome of intact mitochondria by cross-linking mass spectrometry provides evidence for coexisting respiratory supercomplexes
Source: Mol Cell Proteomics. 2017 Dec 8;17(2):216–32. doi: 10.1074/mcp.RA117.000470 (PMC5795388; doi:10.1074/mcp.RA117.000470)
Supplement: Supplemental Data [file supp_RA117.000470_133922_0_supp_23978_fzffwf.zip › spectra_annotation/mito_spectra_annotation/407-1-1-1-11-1.pdf]

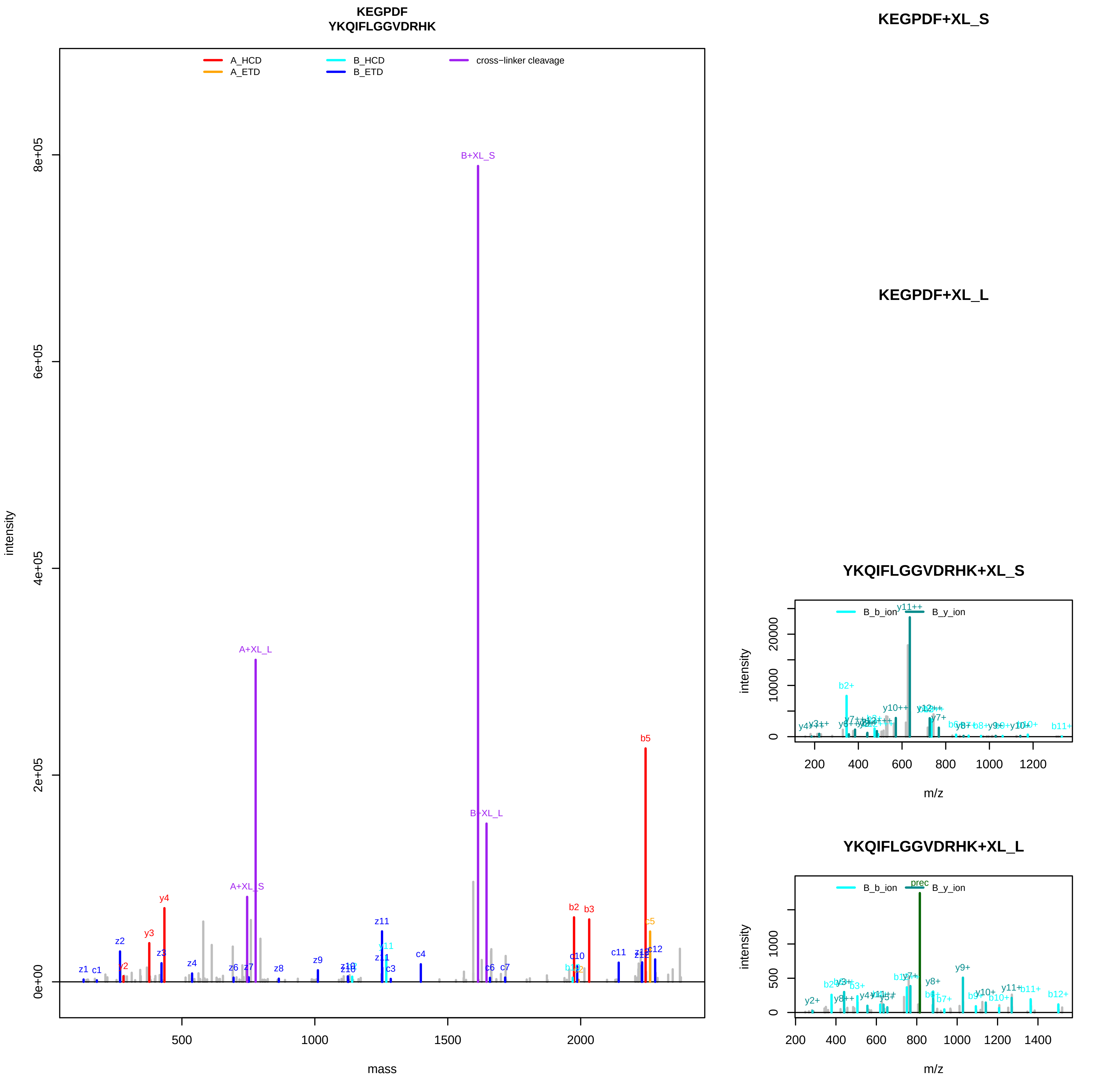
KEGPDF
YKQIFLGGVDRHK
A_HCD
A_ETD
B_HCD
B_ETD
cross−linker cleavage
B+XL_S
A+XL_L
A+XL_S
B+XL_L
b5
b2
b3
c5
y4
y3
y2
z1
c1
z2
z3
z4
z6
z7
z8
z9
z10
z11
y11
c3
c4
c6
c7
c10
c11
c12
intensity
mass
0e+00
2e+05
4e+05
6e+05
8e+05
500
1000
1500
2000
KEGPDF+XL_S
KEGPDF+XL_L
YKQIFLGGVDRHK+XL_S
B_b_ion
B_y_ion
y11++
b2+
y10++
y12++
y7+
intensity
m/z
0
10000
20000
200
400
600
800
1000
1200
YKQIFLGGVDRHK+XL_L
B_b_ion
B_y_ion
prec
y9+
y8+
y7+
y3+
b2+
b3+
y2+
y8++
y10+
y11+
b11+
b12+
b10+
b9+
b7+
b6+
intensity
m/z
0
500
1000
200
400
600
800
1000
1200
1400
